# 13-gene DNA Methylation Analysis from Oral Brushing: A Promising Non Invasive Tool in the Follow-up of Oral Cancer Patients

**DOI:** 10.3390/jcm8122107

**Published:** 2019-12-02

**Authors:** Davide B. Gissi, Achille Tarsitano, Andrea Gabusi, Roberto Rossi, Giuseppe Attardo, Jacopo Lenzi, Claudio Marchetti, Lucio Montebugnoli, Maria P. Foschini, Luca Morandi

**Affiliations:** 1 Section of Oral Sciences, Department of Biomedical and Neuromotor Sciences, University of Bologna, 40159 Bologna, Italy; andrea.gabusi3@unibo.it (A.G.); roberto.rossi30@studio.unibo.it (R.R.); lucio.montebugnoli@unibo.it (L.M.); 2 Section of Maxillo-Facial Surgery at Policlinico S. Orsola-Malpighi, Department of Biomedical and Neuromotor Sciences, University of Bologna, 40138 Bologna, Italy; achille.tarsitano2@unibo.it (A.T.); claudio.marchetti@unibo.it (C.M.); 3 Section of Anatomic Pathology at Bellaria Hospital, Department of Biomedical and Neuromotor Sciences, University of Bologna, 40139 Bologna, Italy; giuseppe.attardo@student.umons.ac.be (G.A.); mariapia.foschini@unibo.it (M.P.F.); 4 Section of Hygiene, Department of Biomedical and Neuromotor Sciences, Public Health and Medical Statistics, University of Bologna, 40126 Bologna, Italy; 5 Functional MR Unit, IRCCS Istituto delle Scienze Neurologiche di Bologna, Department of Biomedical and Neuromotor Sciences, University of Bologna, 40139 Bologna, Italy; luca.morandi2@unibo.it

**Keywords:** oral squamous cell carcinoma, bisulfite sequencing, quantitative DNA methylation analysis, oral brushing, follow-up surveillance, prognosis

## Abstract

Background: This study aimed to evaluate the prognostic value of a non-invasive sampling procedure based on 13-gene DNA methylation analysis in the follow-up of patients previously treated for oral squamous cell carcinoma (OSCC). Methods: The study population included 49 consecutive patients treated for OSCC. Oral brushing sample collection was performed at two different times: before any cancer treatment in the tumor mass and during patient follow-up almost 6 months after OSCC treatment, within the regenerative area after OSCC resection. Each sample was considered positive or negative in relation to a predefined cut-off value. Results: Before any cancer treatment, 47/49 specimens exceeded the score and were considered as positive. Six months after OSCC resection, 16/49 specimens also had positive scores in the samples collected from the regenerative area. During the follow-up period, 7/49 patients developed locoregional relapse: 6/7 patients had a positive score in the regenerative area after OSCC resection. The presence of a positive score after oral cancer treatment was the most powerful variable related to the appearance of locoregional relapse. Conclusion: 13-gene DNA methylation analysis by oral brushing may have a clinical application as a prognostic non-invasive tool in the follow-up of patients surgically treated for OSCC.

## 1. Introduction

The survival rate of patients with a diagnosis of Oral Squamous Cell Carcinoma (OSCC) has only slightly improved over the past 20 years, with 5-year survival rates of approximately 60% [1]. The poor survival rate has been ascribed to a high frequency of locoregional recurrence. Indeed, despite aggressive combined-modality treatment regimens, in the aftercare of primary OSCC, a high number of patients risk developing locoregional recurrences (up to 40%) [2], even those with small primary tumors. Moreover, two-thirds of locoregional relapses occur within the first 2 years [3].

It is common practice to enroll patients treated for OSCC in a routine follow-up program. The usual follow-up strategy consists of periodic visual examination and palpation of the oral cavity during the 5-year aftercare period, including examination and palpation of other anatomical subsites of the head and neck region (nasopharynx, oropharynx, hypopharynx, and larynx) [4]. The main goal of a routine follow-up after OSCC is the early detection of recurrence or second primary tumors, together with monitoring functional rehabilitation, psychological support, and quality control [5]. In the case of recurrence, salvage surgery remains the only curative option [6]. Therefore, identification at an early stage of locoregional recurrence is crucial to increase the possibility of prompt curative salvage surgery [7]. Unfortunately, in patients treated for a previous OSCC, post-treatment surveillance may represent a diagnostic challenge. Persistent oral discomfort and tissue distortion from radiation therapy and surgery may obscure or mimic early neoplastic changes. Oral incisional biopsy and histological analysis remain the most reliable procedures to identify an oral carcinoma or a high-risk lesion. However, this is an invasive surgical procedure that is not routinely applied in the follow-up of OSCC-treated patients.

The development and application of non-invasive diagnostic tools are crucial. DNA methylation analysis of oral cells obtained by gently brushing the oral mucosa has recently been demonstrated to disclose early genetic changes that lead to the development of OSCC [8,9,10]. A DNA methylation profile could be used as an efficient molecular tool for the early diagnosis of OSCC [9,10,11,12,13,14,15,16,17,18]. The presence of an altered DNA methylation profile is a common epigenetic event associated with the genesis of OSCC. Apparently normal oral mucosa adjacent to the OSCC and potentially malignant lesions can have aberrant methylation patterns in candidate genes, even in the absence of histological or genetic abnormalities [19].

We recently developed a non-invasive procedure to identify oral carcinomas at an early stage through oral brushing, in which we quantitatively measured the DNA methylation level of a panel of 13 genes [15].

In that study, the methylation profiles of *ZAP70*, *ITGA4*, *KIF1A*, *PARP15*, *EPHX3*, *NTM*, *LRRTM1*, *FLI1*, *MIR193*, *LINC00599*, *MIR296*, *TERT,* and *GP1BB* were analyzed, and a score weighting of the best CpGs from all 13 genes was calculated. The results were highly indicative of the presence of a malignant process: 28 of 29 OSCC (96.6%) and six of six high-grade squamous intraepithelial lesion (HGSIL) (100%) specimens exceeded the threshold value, while no specimens from 65 healthy donors exceeded the threshold value. 

The high accuracy of the method encouraged us to apply this non-invasive tool to a cohort of patients who had previously been treated for OSCC. This study evaluated whether DNA methylation analysis of samples collected through oral brushing could have a prognostic value in the follow-up of patients previously treated surgically for OSCC.

## 2. Experimental Section

### 2.1. Ethics Statement

All clinical investigations were conducted according to the principles expressed in the Declaration of Helsinki. The study was approved by the local Ethics Committee (study number 14092, protocol number 899/CE). All information regarding the human material used in this study was managed using anonymous numerical codes. Each participant gave informed consent.

### 2.2. Population Study

Oral brushing specimens were collected from 49 consecutive patients treated surgically for OSCC. All 49 patients were diagnosed and treated at the Department of Biomedical and Neuromotor Sciences, University of Bologna, Section of Oral Sciences and the Maxillofacial Surgery Unit, Sant’Orsola Hospital during the period 2011–2018. Histological diagnoses were conducted at the Anatomic Pathology Unit at Bellaria Hospital in the same department. Surgical resection of OSCC was always performed in accordance with standard treatment practice [20]. In 26 of 49 surgically-treated patients, a reconstruction using local or microvascular free flaps was performed in relation to the disease stage. Moreover, 27 of 49 patients received adjuvant radiotherapy.

Selection criteria were as follows: complete surgical resection with no histologically-involved margins of OSCC; and no clinical or radiographic evidence of relapse within 6 months of the end of treatment.

Clinical follow-up of this cohort of patients was performed every 2 weeks for the first 2 months after surgery and then monthly during the first year after surgery, every 3 months during the second year after surgery, and finally every 6 months. A CT scan or MRI was requested every 6 months during the first 3 years after surgery and then once a year. During the follow up period, clinical, instrumental and radiological examinations were administered according to international National Comprehensive Cancer Network (NCCN) guidelines. 

Oral brushing sample collection was performed in the population study as previously described at two different times [10,15,21]:

(1) Before any cancer treatment, to evaluate the presence of an altered methylation pattern in the tumor mass; and (2) during patient follow-up almost 6 months after OSCC treatment, within the regenerative area after OSCC resection (with or without the presence of a reconstructive tissue transfer used for the surgical repair after OSCC resection). We included in the study population only patients that showed an apparently healthy mucosa without the presence of a suspected neoplastic and/or preneoplastic lesion in the regenerative area. Brushing specimens were collected at the Department of Biomedical and Neuromotor Sciences, University of Bologna, Section of Oral Sciences and the Maxillofacial Surgery Unit, Sant’Orsola Hospital from 2014 to 2018.

Clinical and histological information related to the index OSCC of patients included: age; sex; location of primitive tumor; tumor stage according to p-TNM classification of tumors (AJCC 8th edition) [22]; clinically positive cervical lymph node metastasis (LNM) at tumor presentation using criteria defined in van den Brekel et al. [23]; the presence or absence of a concurrent oral potentially malignant disorder in the oral cavity (diagnosed as Oral Leukoplakia or Oral Lichen Planus); the presence or absence of perineural invasion; the presence or absence of vascular invasion; histological grade in agreement with Kademani et al. [20]; and status of surgical margin assessed at the closest point to the surgical resection margin and classified in four categories according to the guidelines of the Royal College of Pathologists in the United Kingdom [24]: *clear*—no evidence of microscopic carcinoma or presence of dysplasia within 5 mm of the margin; *close*—histological evidence of carcinoma between 5 and 1 mm of the margin but not at the margin; (3) *Presence of moderate-to-severe dysplasia* or in situ carcinoma but not invasive carcinoma within 5 mm of the margin; (4) *involved*: evidence of microscopic carcinoma within 1 mm of the margin. In our cohort of study, 45/49 OSCC patients showed clear surgical margins, 3 patients showed close surgical margins, and 1 patient showed the presence of dysplasia within 5 mm of the margin. The presence of a surgical margin involved resulted in exclusion from the present study.

Disease-free survival endpoints, defined as the duration between OSCC histological confirmation and the appearance of locoregional neoplastic manifestation (local recurrence, second primary tumor, and/or lymph node metastasis) or the last follow-up visit were evaluated in May 2019.

### 2.3. DNA Methylation Analysis

DNA methylation analysis was evaluated as previously described by Morandi et al. [15]. In brief, DNA from exfoliating brush specimens was purified using The MasterPure™ Complete DNA Purification Kit (Lucigen, Middleton, WI, USA, cod. MC85200) and treated with sodium bisulfite using the *EZ*DNA Methylation-Lightning™ Kit (ZymoResearch, Irvine, CA, USA, cod. D5031) according to the manufacturer’s instructions. Quantitative DNA methylation analysis was performed by next-generation sequencing for the following genes: Z*AP70*, *ITGA4*, *KIF1A*, *PARP15*, *EPHX3*, *NTM*, *LRRTM1*, *FLI1*, *MIR193*, *LINC00599*, *MIR296*, *TERT*, and *GP1BB*. Libraries were prepared using the Nextera™ Index Kit (Illumina, San Diego, CA, USA) by a locus-specific bisulfite amplicon approach [25] and loaded onto MiSEQ (Illumina, San Diego, CA, USA, cod. 15027617). FASTQ output files were processed for quality control (> Q30) and converted into FASTA format in a Galaxy Project environment [26]. The methylation ratio of each CpG was calculated in parallel by different tools: BSPAT [27], BWAmeth followed by the MethylDackel tool in a Galaxy Project environment (Europe), EPIC-TABSAT [28], and finally Kismeth [29].

**Figure 1:**
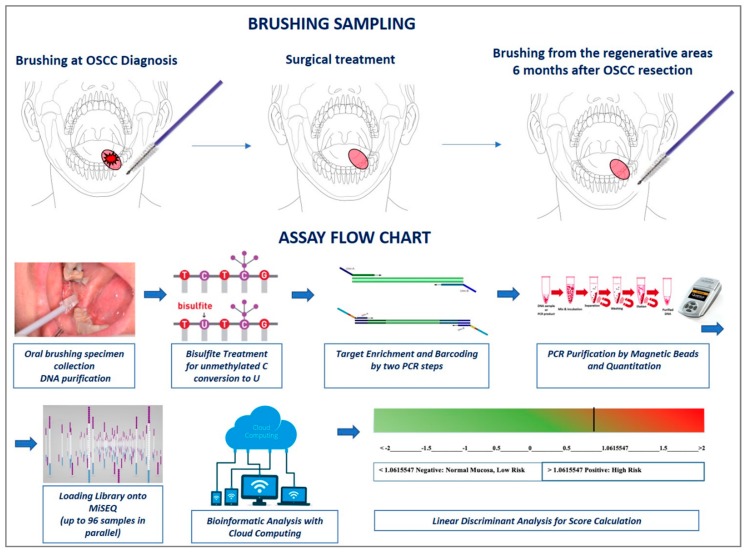
The assay design is based on various steps: (1) In the present study, sampling cell collection was performed at two different moments: before any cancer treatment and during patient follow-up almost 6 months after OSCC treatment; (2) DNA purification and bisulfite treatment (unmethylated cytosines are chemically converted to uracils, while methylated cytosines remained unchanged); (3) Target-specific amplification of 13-gene panel (*ZAP70, ITGA4, KIF1A, PARP15, EPHX3, NTM, LRRTM1, FLI1, MIR193, LINC00599, MIR296, TERT*, and *GP1BB*) and barcoding using Nextera™ index kit (Illumina); (4) PCR purification, quantification and pooling; (5) loading onto MiSEQ; (6) DNA methylation level of a series of 243 CpGs representatives of the 13-gene promoters was calculated in cloud computing using different web tools: BSPAT, BWAmeth, MethylDackel, Kismeth and finally EPIC-TABSAT; (7) score calculation: An algorithm of choice was used to calculate the final score of each sample. This was done multiplying the DNA methylation level of the most informative CpGs previously identified for the corresponding coefficient and adding the constant (see Morandi et al. for details [15]).

In the previous study [15], the best CpGs identified by ROC analysis were used to generate a choice algorithm based on a multiclass linear discriminant analysis. This allowed us to correctly identify OSCC with a threshold of 1.0615547 as the best value for sensitivity and specificity (AUC = 0.981). Using the same algorithm, in the present study, a specific score was calculated for each sample. Values exceeding the threshold of 1.0615547 were considered positive (Figure 1).

### 2.4. Statistical Analysis

Each sample was analyzed either as a numeric or a dichotomous variable (positive/negative) in statistical analyses. Chi-square analysis and one-way analysis of variance (ANOVA) were used to compare score samples obtained in the regenerative area after tumor resection with respect to age, sex, smoking habits, T-stage of OSCC, presence or absence of N-positivity at OSCC presentation, presence of concomitant OPML, histological grade of OSCC, localization of index OSCC, presence or absence of perineural invasion, and presence or absence of vascular invasion. 

Locoregional disease-free survival was the desired outcome in the present study. One way ANOVA analysis was used to evaluate differences between the mean score values in patients who experienced locoregional neoplastic manifestation after oral brushing collection and patients free of disease, for the specimens collected from the regenerative area after OSCC resection. 

Positive scores in the regenerative area after OSCC resection were analyzed for any relationship with outcome. Survival rate was estimated using the Kaplan–Meier method. Statistical significance was evaluated using the log-rank test. Time was defined as the period between OSCC histological confirmation and appearance of locoregional neoplastic manifestation or the last follow-up visit. Statistical analysis also evaluated the role of potential confounding variables related to the OSCC index and patient age, sex, smoking habits, T stage of OSCC, presence or absence of N positivity at OSCC presentation, presence of concomitant OPML, histological grade of OSCC, localization of index OSCC, presence or absence of perineural invasion, and the presence or absence of vascular invasion. For those variables found to be statistically significant at univariate analysis with a significance level of *p* < 0.25, the Cox proportional hazards method with stepwise bidirectional selection (significance level of entry/removal = 0.05) was used for further evaluation by multivariate survival analysis. The proportionality of hazards assumption of the model was tested on the basis of Schoenfeld’s test. *p* values < 0.05 were considered statistically significant in all analyses.

## 3. Results

The study population comprised 30 women and 19 men aged 41–86 years with a mean age at the index neoplastic event presentation of 67.27 ± 11.56 years. Seventeen tumors were on the tongue, 1 on the floor, 4 in the cheek, 7 in the upper gingival and hard palate or both, 19 in the lower gingival, and 1 on the lip. 

### 3.1. Gene DNA Methylation Analysis

A series of 10 samples were processed in duplicates to evaluate the reliability of the assay with linear regression analysis (R^2^: 0.8392; *p* = 0.0002). The same threshold of 1.0615547, adopted for diagnostic purposes described by Morandi et al. [15], was considered to stratify patients. 

#### 3.1.1. Before Surgery

A mean score value of 2.78 ± 1.48 was calculated for the group of 49 oral brushing specimens collected at OSCC diagnosis. A total of 47 of the 49 (95.9%) specimens exceeded the threshold value and were considered positive. 

#### 3.1.2. After Surgery

**Figure 2:**
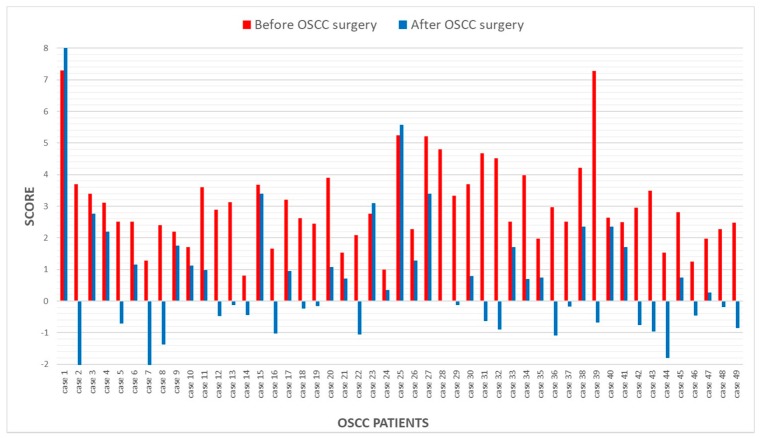
Bar chart obtained using the scores calculated from the algorithm for each patient: Red columns represent scores of brushing samples collected at OSCC diagnosis whereas blue columns show scores of brushing samples collected from the regenerative area 6 months after OSCC resection.

A mean score value of 0.63 ± 2.05 was calculated for the group of 49 oral brushing specimens from the regenerative area after OSCC resection. A total of 16 of the 49 (32.6%) specimens exceeded the threshold value and were considered positive. Patients that showed a negative score at the time of OSCC diagnosis also had a negative value for the 13-gene DNA methylation analysis in the brushing sample collected during the follow-up period (Figure 2).

None of the clinicopathological variable results were related to the score calculated for the regenerative areas. Specifically, no significant difference in the methylation profile for the regenerative area after OSCC resection (*p* = ns) was found in patients who underwent adjuvant therapy and patients who did not. 

### 3.2. Loco-Regional Disease-Free Survival

The follow-up period ranged from 3 to 72 months (mean follow-up period, 18.9 ± 17.6 months). Seven of 49 (14.3%) patients developed a neoplastic manifestation during follow-up; one patient developed a lymph node metastasis as a secondary neoplastic manifestation, five patients developed a recurrence at the site of the index tumor (<2 cm), and one patient developed a secondary tumor at a different site in the oral cavity with respect to the index tumor (>2 cm). Table 1 lists the clinical, histological, and epigenetic characteristics related to brushing specimens of the seven patients who developed a secondary neoplastic manifestation.

Six patients who developed a second neoplastic manifestation showed a positive score in brushing samples collected from the regenerative areas 6 months after OSCC resection and only one patient with a negative test in the post-treatment brushing sample developed lymph node metastasis as a secondary neoplastic manifestation. 

In the group of patients with no recurrence, 10 had positive scores and 32 had negative scores in samples collected from the regenerative areas.

**Figure 3:**
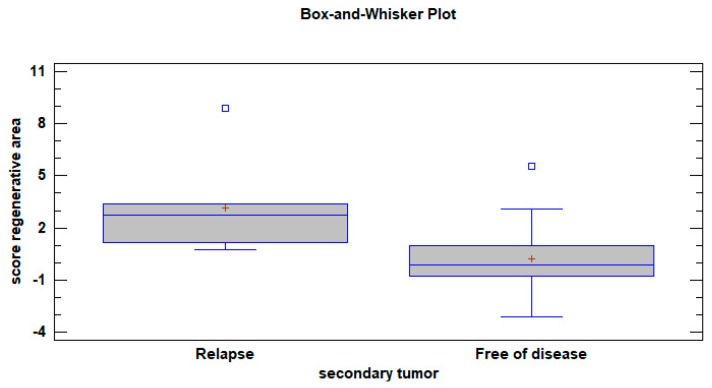
Box-plots obtained using the scores calculated from the algorithm showed a significant between-group difference (One way ANOVA F = 17.17; *p* < 0.0001).

Analysis of variance indicated that patients who experienced a secondary tumor during the follow-up period showed significantly higher numeric values in the specimens collected in the regenerative areas after OSCC resection (F = 17.17; *p* < 0.0001) (Figure 3).

**Figure 4:**
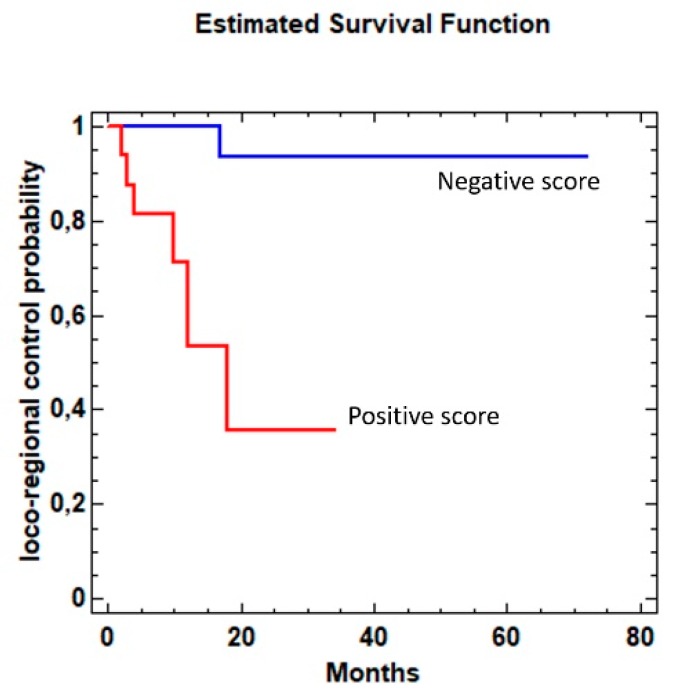
Kaplan Meier estimate for disease-free survival rate by 13-gene DNA methylation analysis in oral brushing samples collected from the regenerative areas 6 months after cancer resection. Significantly worse LRC (*p* < 0.01) was found for patients with a positive score.

Kaplan–Meier analysis revealed that a positive test result in samples collected from the regenerative areas after cancer resection was significantly related to worse loco-regional control (LRC) of the disease (Chi 13.5152; *p* < 0.0001) (Figure 4).

Results of the Kaplan–Meier analysis also showed that the presence of perineural (Chi 12.16; *p* < 0.01) and vascular invasion (Chi 19.815; *p* < 0.05) resulted in histological variables significantly related to a worse LRC of the disease. Three out of seven patients with perineural infiltration and two out of three patients with vascular infiltration in the OSCC mass developed a subsequent neoplastic event compared with four out of 41 patients with no perineural infiltration and five out of 45 patients with no vascular infiltration. Table 2 lists the characteristics of the study population in terms of score samples from the regenerative area after OSCC resection and LRC.

The variables considered for inclusion in the final multivariate Cox model of LRC were sex, age, grading, site, tumor stage, perineural invasion, vascular invasion, and positive score in regenerative area (*P* < 0.25). The stepwise model selection showed that the presence of positive scores in the regenerative area after OSCC resection and perineural invasion were the most powerful prognostic factors.

Multivariate analysis showed that the presence of positive scores in the regenerative area after OSCC resection and perineural invasion were the most powerful prognostic factors (Table 3).

The proportionality of hazards assumption of the model was tested using the global test. The model was not significant based on the Schoenfeld’s test (*p* = 0.94) indicating that the data did not violate the proportional hazards assumption. Df: Degrees of freedom.

## 4. Discussion

Local relapse occurs in up to 40% of cases of OSCC patients following surgical treatment [2] and is the major factor impacting their survival rate. It is well known that second tumor manifestations follow two distinct pathogenic mechanisms. Slaughter (1953) introduced the concept of field cancerization (also known as field effect) in which he suggested that OSCC is often surrounded by cells that present tumor-related genetic alterations such that the development of secondary neoplasia is a consequence of additional genetic hits within this field, following a multistep process [30]. On the other hand, a local relapse may be related to the possibility of an incomplete cancer resection. Different mechanisms have been proposed to explain the presence of residual malignant cells even in presence of tumor-free resection margins, such as shedding of malignant cells into the saliva and implantations at other sites or lateral migration of isolated malignant cells [2,31]. The routine use of non-invasive molecular prognostic tests in the post-treatment surveillance of OSCC patients presents an attractive strategy to identify patients presenting genetic and epigenetic alterations or both, even in the regenerative mucosa after surgery that could determine a significant risk of developing into secondary oral cancer. 

The presence of genetically-altered cells in the surgical margins and tumor-adjacent normal tissue has been shown by molecular analyses [32]. Most studies have identified genetic alterations at the surgical margins in formalin-fixed paraffin-embedded (FFPE) samples but also in extra-biopsies and brushed cells taken from tumor-adjacent clinically- and histologically-normal mucosa or both [33,34,35]. Our previous studies using Ki67 expression to evaluate abnormally high cell turnover demonstrated that a percentage of OSCC patients show abnormally high cell turnover in the clinically- and histologically-normal distant mucosa located far from the primary tumor (e.g., on the opposite cheek) [36,37,38].

Epigenetic silencing events (i.e., promoter hypermethylation) are frequent events in oral carcinogenesis and can precede genetic alterations and changes in protein expression. Recently, the presence of an altered methylation profile in the surgical margins and tumor-adjacent normal tissue was identified in fixed tissue and fresh clinical samples [8,39]. Recently, we developed a new assay based on DNA methylation analysis of a set of 13 genes, which were able to detect precisely the presence of OSCC independently from the region in the oral cavity where the carcinoma occurred [15]. In fact, we enrolled in that previous study 29 OSCC for the training dataset, of which eight came from the tongue, four from the floor of mouth, eight from gum, five from cheek, three from palate, and one from lip. The sensitivity of the assay was 97.1%, and this performance was confirmed in the current study identifying a positive score in 47 out of 49 OSCC cases collected by brushing at diagnosis prior to surgical removal (Sensitivity: 95.9%). These 13 selected genes were previously described to be involved in OSCC [10,15,40] and were found to have a crucial role also in other malignancies such as for instance ZAP70 in chronic lymphocytic leukemia [41]; TERT in all the most common tumor types [42]; and FLI1 in Ewing sarcoma, rectal cancer and gastric cancer [43]. This probably justifies the ability of these restricted number of genes to be able to clearly detect cancer cells despite the tissue type they come from (cheek, tongue or hard palate).

The present study was designed for prognostic purposes to determine whether our procedure based on DNA methylation analysis of a panel of 13 genes starting from a non-invasive sampling procedure could identify an altered methylation profile even in clinically healthy mucosa that had replaced the surgical areas after OSCC removal. In the case of a positive score, a related high risk of relapse was evaluated.

Interestingly, 13-gene DNA methylation analysis on brushing samples collected 6 months after OSCC treatment showed that 16 of 49 (32.6%) samples from the regenerative area after tumor resection exceeded the threshold value and were considered positive. 

Recently, an altered methylation profile was also identified in a percentage of post-operative samples from OSCC patients, based on non-invasive sampling procedures (swab and saliva). Different gene markers and approaches to characterize the methylation pattern were used in these studies (e.g., methylation array analysis and quantitative methylation-specific PCR) [16,18,44].

It remains to be clarified whether the identification of an altered methylation profile, determined by a positive score, in the oral mucosa of previously-treated OSCC patients, is a viable assay to identify patients at risk of developing secondary neoplasia. It is well-accepted that methods for promoter methylation detection are more reliable when fresh clinical samples rather than FFPE tissues are used [8], and this is why the development of a non-invasive molecular tool for the post-treatment surveillance is of utmost importance.

Meanwhile, the potential use of DNA methylation analysis of one or a combination of genes has been investigated as a prognostic marker in head and neck squamous cell carcinoma based on non-invasive sampling collection [9,11,13,14,16,18,44,45,46,47,48]; however, the majority of these studies found that an altered methylation level was related to poor prognosis and the samples were collected at the time of OSCC diagnosis (pretreatment). The prognostic value of promoter hypermethylation was identified for one gene in a pattern of genes from post-operative saliva samples. In particular, Righini et al. collected saliva samples every 2 to 6 months post-diagnosis and treatment and showed that hypermethylation of five genes (*TIMP3*, *ECAD*, *p16*, *MGMT*, *DAPK*, and *RASSF1A*) was associated with relapse in OSCC patients [44]. Rettori et al. collected saliva samples immediately after the last curative treatment and at a follow-up visit 6 months after treatment in which *TIMP3* promoter hypermethylation was detected in the post-treatment salivary rinse as an independent prognostic marker for local recurrence-free survival in patients with head and neck cancer [16]. 

In our study, seven of 49 patients developed secondary neoplasia during the follow-up period, and six had a positive score in the regenerative area after OSCC resection. Multivariate analysis revealed that this parameter was the most powerful independent variable, even before perineural invasion, and these data suggest that our procedure may have a clinical application in the early detection of secondary events.

In particular, five patients with a positive score for samples collected in the regenerative area after OSCC resection developed a secondary tumor at the same site of the index tumor, suggesting that a field effect and small clusters of residual tumor cells that were undetectable in the histological analysis of the margin or both may have proliferated, and thus formed the basis of recurring cancer. Additionally, epigenetic modifications are usually present in the cancerization field, a phenomenon not usually correlated with morphological changes clearly detectable by histology; in this case, the ability to monitor the DNA methylation variations by a non-invasive technique may help to predict recurrences. One patient developed a secondary tumor (left tongue) at a different site with respect to the index tumor (right tongue), suggesting the presence of a wider field effect. 

One patient with locoregional relapse and who had a negative score in the post-treatment brushing sample developed an extraoral secondary neoplastic manifestation (lymph node metastasis) 7 months after the index tumor in the oral cavity. A potential explanation is that lymph node metastasis represented a delayed clinical manifestation of the previously-treated primary tumor or cells from the first OSCC that may have spread through the saliva, tissue, lymphatic system, or bloodstream. It is likely that in this situation, a field effect or small clusters of residual tumor cells undetectable in the histological analysis of the margin would have been undetectable in the oral cavity [31]. Further investigations are needed to ascertain whether our procedure is informative for secondary neoplasia that arise in the oral cavity. 

## 5. Conclusions

This prospective study demonstrated that DNA methylation analysis of a 13-gene panel of oral brushing samples revealed the presence of an altered methylation status in clinically non-neoplastic mucosa of patients previously treated for OSCC. Moreover, an altered methylation profile, identified by a positive score, was shown to be an independent prognostic factor that was significantly associated with the appearance of a secondary tumor. These findings suggest that our procedure could be used as an indicator of disease before the appearance of clinical signs and symptoms in surgically-treated OSCC patients and that a positive score may have clinical applications in the future as a surveillance tool or as part of multi-modal therapy for LRC. Further investigations with a larger study cohort, an adequate follow-up period, and with oral brushing sample collection at different times during OSCC patient follow-up are needed to gain a better understanding of the prognostic value of our procedure.

## Figures and Tables

**Table 1:** Clinical, histological, and epigenetic characteristics of the seven patients who developed a secondary neoplastic manifestation during follow-up period.

Patient	Site of Index Tumor	Site of Secondary Tumor	TNM Stage of Index Tumor	Perineural Invasion	Vascular Invasion	Histological Grade	Status of Surgical Margin	Presence of Concomitant OPMD	DNA Methylation Score in OSCC Sample (pre-treatment)	DNA Methylation Score in Regenerative Area (post-treatment)
Case 1	Palate	Palate	T1N0M0	Yes	Yes	G2	Clear	No	7.29 (POSITIVE)	8.88 (POSITIVE)
Case 3	Superior gum/hard palate	Superior gum/hard palate	T2N2bM0	No	No	G2	Clear	No	3.39 (POSITIVE)	2.76 (POSITIVE)
Case 6	Superior gum/hard palate	Superior gum/hard palate	T1N0M0	No	No	G2	Close	Lichen	2.51 (POSITIVE)	1.15 (POSITIVE)
Case 15	Right tongue	Left tongue	T2N3M0	Yes	No	G2	Clear	No	3.68 (POSITIVE)	3.39 (POSITIVE)
Case 27	Superior gum/palate	palate	T2N0M0	Yes	Yes	G3	Clear	No	5.21 (POSITIVE)	3.38 (POSITIVE)
Case 33	Hard palate	Hard palate	T1N0M0	No	No	G1	Clear	Lichen	2.51 (POSITIVE)	1.69 (POSITIVE)
Case 35	Right Cheek	Lymph node metastasis	T3N0M0	No	No	G3	Clear	No	1.97 (POSITIVE)	0.73 (NEGATIVE)

TNM: American Joint Committee on Cancer (AJCC) staging system; OPMD: Oral Potentially Malignant Disease; OSCC: Oral Squamous Cell Carcinoma.

**Table 2:** The study population characteristics in terms of score samples from the regenerative area after OSCC resection and Loco-Regional Control (LRC). Entries in bold format and withasterisk (*) indicate statistically significant *p* values (*p* < 0.05) of prognostic variables related to appearance of a loco-regional secondary neoplastic manifestation during follow-up period.

Clinico-Pathological Variables	Patients	Positive Score Regenerative Mucosa	*p*	Relapse (LR, SPT or LNM)		
				N Patients	% Patients	*p*
**Sex**						
Male	19	4	*p* = 0.07	1	5.3%	*p* = 0.06
Female	30	12		6	20%	
**Age**						
< 65	18	4	*p* = 0.234	1	5.5%	*p* = 0.07
> 65	31	12		6	19.5%	
**Site**						
Tongue	17	3	*p* = 0.07	1	5.9%	*p* = 0.07
Floor of mouth	1	0		0	0	
Cheek	4	0		1	25%	
Soft palate	0	0		0	0	
Superior gum/hard palate	7	5		2	28.6%	
Inferior gum	19	8		3	15.8%	
Lip	1	0		0	0	
**Grading**						
G1	20	7	*p* = 0.37	1	5%	*p* = 0.06
G2	21	8		6	28.6%	
G3	8	1		0	0	
**T stage**						
T1–2 = 0	28	9	*p* = 0.93	6	21.4%	*p* = 0.08
T3–4 = 1	21	7		1	4.7%	
**N positivity**						
N0	42	14	*p* = 0.81	6	14.3%	*p* = 0.93
N+	7	2		1	14.3%	
**Perineural invasion**						
No	42	13	*p* = 0.53	4	9.5%	*p* = 0.0004 *
Yes	7	3		3	42.8%	
**Vascular invasion**						
No	46	13	*p* = 0.06	5	10.9%	*p* = 0.000006 *
Yes	3	3		2	66.7%	
**Margin of resection tumor**						
Clear	45	14	*p* = 0.35	6	13.3%	*p* = 0.48
Close	3	2		1	33.4%	
Dysplasia	1	0		0	0	
**Presence of OPMD**						
No	42	13	*p* = 0.26	5	11.9%	*p* = 0.38
Oral Lichen Planus	5	3		2	40%	
Leukoplakia	2	0		0	0	
**Adjuvant Radiotherapy**						
No	27	9	*p* = 0.91	3	11.1%	*p* = 0.58
Yes	22	7		4	18.2%	
**Reconstructive flap**						
No	23	7	*p* = 0.75	3	13.1%	*p* = 0.73
Yes	26	9		4	15.4%	
**Score in regenerative area**						
Negative	33			1	3.1%	*p* = 0.0002 *
Positive	16			6	37.5%	

Entries in bold format and with asterisk (*) indicate statistically significant *p* values (*p* < 0.05) of prognostic variables related to appearance of a loco-regional secondary neoplastic manifestation during follow-up period.

**Table 3:** Statistically significant variables by multivariate analysis for predicting loco regional disease-free survival.

Factor	Df	Hazard Ratio	*p-*Value
Perineural invasion	1	7.84 (1.28–48.12)	0.0279
Positive score in regenerative area	1	15.02 (1.7–130.2)	0.0024
